# Effects of Two Dental Implant Micromotor Systems for Dental Implant Placement on Implant Stability and Removal Torque: An Animal Experiment

**DOI:** 10.3390/ma18174048

**Published:** 2025-08-29

**Authors:** Keunbada Son, Young-Tak Son, Sung-Min Hwang, Jae Mok Lee, Jin-Wook Kim, Kyu-Bok Lee

**Affiliations:** 1 Advanced Dental Device Development Institute (A3DI), Kyungpook National University, Daegu 41940, Republic of Korea; oceanson@knu.ac.kr (K.S.); dudxkr741@naver.com (Y.-T.S.); 2 Department of Dental Science, Graduate School, Kyungpook National University, Daegu 41940, Republic of Korea; 3 Department of Periodontology, School of Dentistry, Kyungpook National University, Daegu 41566, Republic of Korea; dent_hwang@knu.ac.kr (S.-M.H.); leejm@knu.ac.kr (J.M.L.); 4 Department of Oral & Maxillofacial Surgery, School of Dentistry, Kyungpook National University, Daegu 41566, Republic of Korea; vocaleo@knu.ac.kr; 5 Department of Prosthodontics, School of Dentistry, Kyungpook National University, Daegu 41940, Republic of Korea

**Keywords:** dental implant micromotor, implant stability quotient, resonance frequency analysis, bone–implant interface gap, micro-computed tomography, removal torque

## Abstract

This in vivo animal study aimed to evaluate the effects of two different implant placement micromotor systems on implant stability and removal torque. In a within-animal crossover design, twenty titanium implants (AnyOne fixture; internal type; diameter, 3.5 mm; length, 7.0 mm; Megagen, Daegu, Republic of Korea) were placed in the tibiae of five rabbits using a conventional micromotor system (NSK group: SurgicPro+; NSK, Kanuma, Japan) and a diode laser-integrated micromotor system (SAESHIN group: BLP 10; Saeshin, Daegu, Republic of Korea). Resonance frequency analysis provided the implant stability quotient (ISQ) immediately after placement and at four weeks. Micro-computed tomography quantified the bone–implant interface gap (BIG). Removal torque was measured at sacrifice. Linear mixed-effects models with a random intercept for rabbit generated adjusted means with 95% confidence intervals (CIs) (α = 0.05). Equivalence for the four-week ISQ used two one-sided tests with a margin of ±5 ISQ. The SAESHIN group achieved a higher immediate ISQ than the NSK group (difference =+6.9 ISQ; 95% CI +1.3–+12.5; *p* = 0.018). At four weeks, the ISQ did not differ (difference = −1.2 ISQ; 95% CI −4.3–+1.9; *p* = 0.42), and equivalence was supported (TOST *p*_lower = 0.024; *p*_upper = 0.019). Removal torque was comparable (difference = +4.3 N·cm; 95% CI −5.2–+13.8; *p* = 0.36). BIG metrics showed no between-system differences across regions. ICC indicated clustering for ISQ and torque (0.36 and 0.31). The diode laser-integrated micromotor system yielded a higher immediate ISQ under a standardized 35 N·cm seating torque, whereas the ISQ, removal torque, and BIG at four weeks were comparable to those of the conventional system. The immediate ISQ should be interpreted as stiffness under fixed torque rather than superior device-dependent interlocking. These findings support the clinical interchangeability of the two systems for early osseointegration endpoints in preclinical settings.

## 1. Introduction

Dental implants have become a widely accepted and highly predictable modality for tooth replacement, demonstrating long-term success rates exceeding 95% over a 10-year period [1,2,3]. The functional longevity of dental implants is fundamentally dependent on achieving and maintaining adequate primary stability during the initial healing phase, a period that critically influences osseointegration outcomes [4,5]. Primary stability refers to the mechanical interlocking of the implant within the surrounding bone at the time of placement and is primarily affected by variables such as surgical technique, bone density, implant geometry, and drilling instrumentation [6,7,8]. Inadequate primary stability can result in micromotion at the bone–implant interface, promoting fibrous tissue encapsulation, delayed osseointegration, or outright implant failure [9,10]. Conversely, excessive insertion torque or thermal injury caused by drilling can compromise peri-implant bone viability and impede osseointegration [11,12,13].

Despite its importance, the influence of surgical micromotor systems on implant placement and primary stability remains underrecognized. Traditional implant motors are susceptible to torque degradation following repeated autoclave sterilization cycles, introducing variability into clinical outcomes [14,15]. To address these limitations, advanced micromotor systems have been developed. Some platforms integrate a diode laser module for soft-tissue incision, while the micromotor provides controlled torque delivery for osteotomy [16,17]. These laser-integrated micromotor systems may offer workflow and ergonomic advantages for soft-tissue management while maintaining controlled torque delivery during osteotomy [18,19]. However, few studies have rigorously compared the clinical performance and equivalence of these next-generation and conventional micromotor systems under controlled conditions. In the present study, the integrated diode laser was used only for soft-tissue incision. Osteotomy and implant seating were performed with conventional drilling under continuous external saline irrigation; laser assistance for drilling or thermal modulation was neither used nor evaluated.

Growing evidence suggests that successful implant integration results from the dynamic interplay between surgical instrumentation and biological modulation of peri-implant healing. Various surface modification strategies, including enhancement of titanium surface wettability, hydroxyapatite blasting, and plasma-activated calcium-incorporated coatings, have been shown to accelerate early bone response [20,21,22,23]. In addition, adjunctive biological interventions, such as photobiomodulation and low-intensity pulsed ultrasound, have demonstrated potential in promoting early osseointegration and increasing implant stability [24,25,26]. These findings underscore the multifactorial nature of implant integration and highlight the importance of optimizing both surgical techniques and biological strategies.

To assess implant stability quantitatively, resonance frequency analysis (RFA) is widely recognized as a non-invasive and reproducible method, yielding standardized implant stability quotient (ISQ) values that reflect micromotion at the bone–implant interface [27,28]. Complementary imaging modalities, particularly micro-computed tomography (micro-CT) in conjunction with three-dimensional (3D) deviation analysis, provide high-resolution evaluation of the bone–implant interface gap (BIG), facilitating detailed assessment of the implant–bone interface and its adaptation over time [29,30,31]. Together, these techniques allow for robust examination of the biomechanical and structural outcomes associated with different implant placement approaches.

In light of these considerations, the present study aimed to conduct a controlled, in vivo comparison of two surgical micromotor systems: a conventional micromotor system (NSK SurgicPro+) and a diode laser-integrated micromotor system (Saeshin BLP 10) with the laser used exclusively for soft-tissue incision. The investigation focused on evaluating the respective effects of these systems on primary and secondary implant stability, peri-implant bone adaptation, and mechanical retention using a standardized rabbit tibia model. By integrating longitudinal ISQ monitoring, high-resolution micro-CT-based 3D analysis of BIG, and quantitative removal torque testing, this study sought to determine whether surgical micromotor selection exerts a measurable influence on early osseointegration outcomes.

## 2. Materials and Methods

### 2.1. Experimental Preparation

This animal study was approved by the Institutional Animal Care and Use Committee (IACUC) of the Daegu-Gyeongbuk Medical Innovation Foundation on 23 December 2022 (Approval No. KMEDI-22090202-00). A total of five adult rabbits were included in the experiment. General anesthesia was induced via intramuscular injection of ketamine (35 mg/kg) and Rompun (5 mg/mL), followed by maintenance with inhalational isoflurane (1–2%). After shaving the proximal tibial region, a skin incision was performed using a diode laser in continuous-wave mode at 1.5 W and 980 nm (BLP 10; Saeshin, Daegu, Republic of Korea).

**Figure 1:**
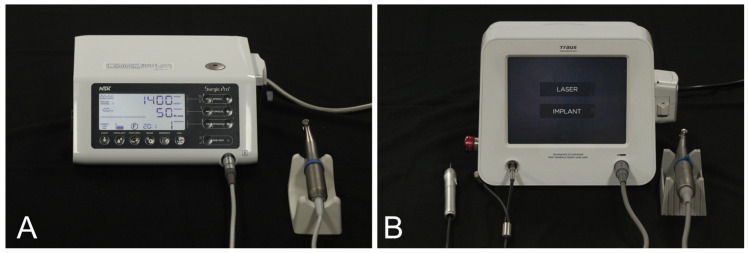
Implant placement micromotor systems used in this study. Each system comprises a surgical micromotor and a corresponding control unit from the same manufacturer, used to regulate torque and rotational speed during implant placement. (**A**) NSK group: SurgicPro+ system (NSK, Kanuma, Japan). (**B**) SAESHIN group: BLP 10 system (Saeshin, Daegu, Republic of Korea), which integrates an implant motor and diode laser within a touchscreen interface for procedural mode selection.

Two distinct surgical micromotor systems were used for implant placement (Figure 1). The control group (NSK group) utilized the SurgicPro+ system (NSK, Kanuma, Japan), while the experimental group (SAESHIN group) employed the BLP 10 system (Saeshin, Daegu, Republic of Korea), which integrates an implant motor with a diode laser module and features a touchscreen interface for operational mode selection.

Initial osteotomy was performed using a pilot drill, followed by sequential enlargement with 2.8 mm and 3.3 mm shaping drills. Commercially available titanium implants (AnyOne; internal connection, Ø3.5 mm × 7.0 mm; Megagen, Daegu, Republic of Korea) were placed into the prepared sites using the designated micromotor system and torque ratchet for each group. Implant stability was assessed immediately following placement using a RFA device. Cover screws were then secured, and the surgical wounds were closed using sutures. Postoperative disinfection was performed with povidone–iodine, and an Elizabethan collar was applied to prevent self-inflicted injury. All animals were maintained under standard laboratory conditions for four weeks prior to sacrifice.

**Figure 2:**
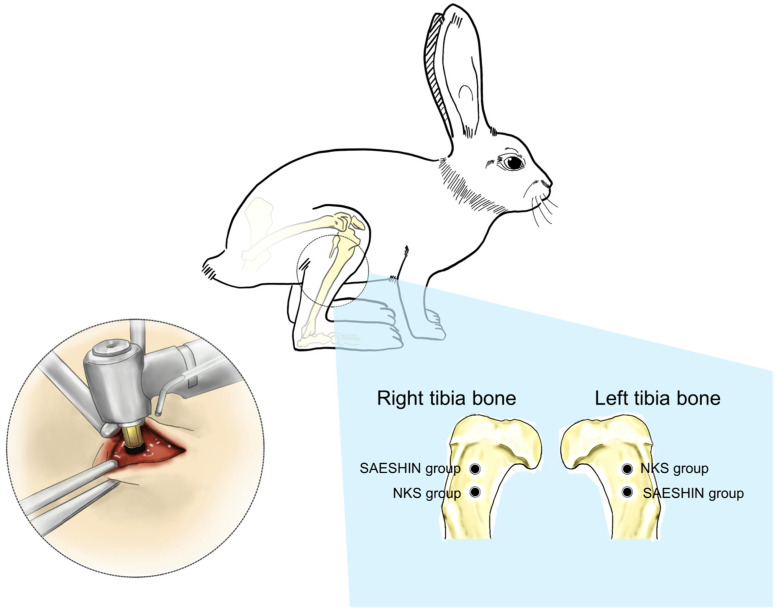
Schematic illustration of implant placement sites in the rabbit tibiae. Implants were placed in the proximal tibiae of both hind limbs using a crossover design to minimize anatomical bias. Each rabbit received two implants per leg, with the NSK and SAESHIN groups alternately assigned to the right and left tibiae. Rabbits (*n* = 5); implants (*n* = 20; NSK *n* = 10, SAESHIN *n* = 10); four per rabbit (two per proximal tibia).

To minimize anatomical variability and control for intra-animal differences, a crossover design was implemented for implant placement in the tibiae of each rabbit (Figure 2). Two implants were placed in each proximal tibia—one assigned to the NSK group and the other to the SAESHIN group—resulting in four implants per rabbit. Implant allocation was alternated between the right and left legs to distribute any limb-related bias. Specifically, in half of the rabbits, the SAESHIN implants were positioned anteriorly in the right tibia and posteriorly in the left tibia, with the NSK implants placed in the opposite configuration. In the remaining rabbits, the positions were reversed to further reduce positional bias. All implant sites were prepared in the metaphyseal region of the proximal tibia to ensure consistent cortical bone engagement and uniform bone quality. This standardized protocol enabled within-subject comparisons between the two surgical micromotor systems, thereby enhancing the statistical robustness and reproducibility of the study findings.

**Figure 3:**
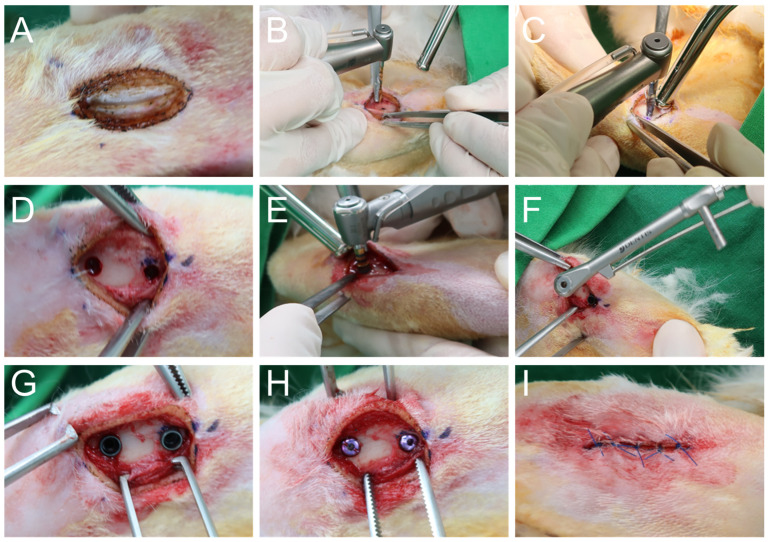
Surgical procedure for implant placement in the rabbit tibia. (**A**) The proximal tibial surface was exposed via surgical incision. (**B**–**D**) Sequential osteotomy was performed using the designated micromotor systems. (**E**–**G**) Implants were inserted using the respective micromotors and finalized with a digital torque gauge. (**H**) Cover screws were connected. (**I**) The surgical site was sutured to complete the procedure.

Under general anesthesia and sterile conditions, all surgical procedures were conducted on the proximal tibiae of both hind limbs in supine-positioned rabbits (Figure 3). Following trichotomy and antiseptic preparation, a linear skin incision approximately 2 cm in length was created using a diode laser in continuous-wave mode (1.5 W, 980 nm) to expose the tibial metaphysis (Figure 3A). Blunt dissection of the underlying musculature was performed to fully expose the bone surface while minimizing soft tissue trauma. Sequential osteotomies were prepared in accordance with each manufacturer’s protocol using the respective surgical micromotors from the NSK (SurgicPro+) and SAESHIN (BLP 10) systems (Figure 3B–D). Drilling was carried out under continuous irrigation with sterile saline to prevent thermal injury to the bone. The osteotomy axis was maintained perpendicular to the tibial surface to ensure accurate implant trajectory. Implant placement was performed using the designated micromotor system for each group (Figure 3E,F), final seating was standardized at 35 N·cm using a calibrated digital torque wrench to control for torque-related confounding. Immediate RFA measurements were therefore interpreted as implant stiffness under a fixed seating torque rather than device-dependent primary mechanical interlock (Figure 3G). Cover screws were then connected (Figure 3H), and the surgical site was closed using 4–0 non-resorbable nylon sutures applied with an interrupted technique (Figure 3I). Postoperative care included the administration of systemic antibiotics (enrofloxacin, 10 mg/kg) and analgesics (meloxicam, 0.2 mg/kg) for three consecutive days.

All animals were adult New Zealand White rabbits (26–32 weeks of age; 3.0–4.0 kg) acclimatized for at least 7 days before surgery and housed under controlled conditions (20–24 °C, 40–70% relative humidity, 12:12 h light/dark cycle) with ad libitum access to standard chow and water, in compliance with the Guide for the Care and Use of Laboratory Animals and an institutional IACUC protocol. Anesthesia was induced with ketamine (35 mg/kg, intramuscular) and maintained with isoflurane (1–2% in oxygen) via face mask; postoperative analgesia consisted of meloxicam (0.2–1.0 mg/kg, once daily for 3 days) with buprenorphine (0.01–0.05 mg/kg) as needed, and antibiotic prophylaxis was provided with enrofloxacin (10 mg/kg, once daily for 3–5 days). To standardize thermal control during osteotomy, continuous external irrigation with sterile saline was delivered by peristaltic pump at approximately 50 mL/min and 20–22 °C, with the nozzle positioned 1.5–2.0 mm from the bur tip and high-vacuum suction about 2 cm away; drilling followed the manufacturer’s stepwise sequence at 800–1200 rpm, with intermittent pecking to limit heat accumulation, and the digital torque wrench was calibrated on the day of surgery to verify the final 35 N·cm seating step. A within-animal crossover allocation was used such that each proximal tibia received one implant per system with anterior–posterior positions alternated between limbs, and outcome measurements were obtained under anesthesia by an assessor not involved in surgery according to a predefined script. Postoperative monitoring included the daily inspection of wounds and general condition, suture removal on postoperative day 7–10 when healing was satisfactory, documentation of immediate stability at placement and secondary stability at 4 weeks, and euthanasia at study completion under deep anesthesia in accordance with predefined humane endpoint criteria.

### 2.2. Evaluation of Implant Stability Quotient

**Figure 4:**
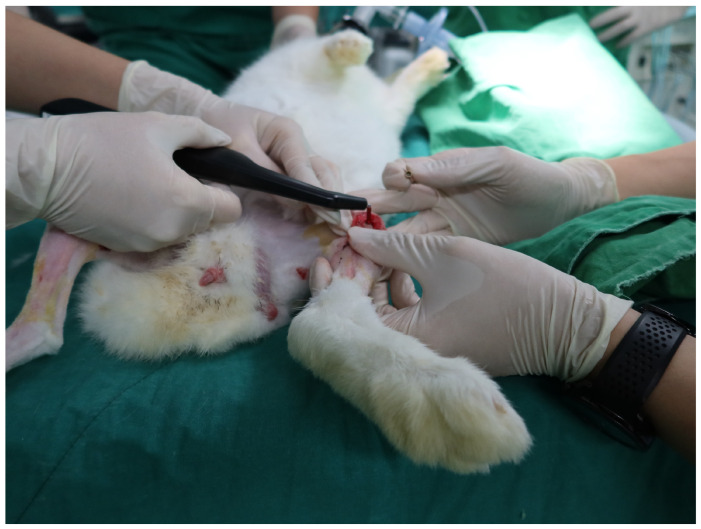
Measurement of implant stability in the rabbit tibia using resonance frequency analysis. Implant stability quotient values were recorded immediately after placement and at 4 weeks postoperatively using a resonance frequency analysis device.

To quantitatively assess the primary and secondary stability of the placed implants, RFA was performed using a non-invasive Osstell Mentor device (Osstell AB, Gothenburg, Sweden), a widely accepted tool for evaluating the stiffness of the bone–implant interface (Figure 4). Measurements were obtained at two time points: immediately following implant placement (primary stability) and at 4 weeks postoperatively, immediately prior to euthanasia (secondary stability). For each measurement, a SmartPeg (type 20, Osstell AB) was attached to the internal connection of the implant and hand-tightened to a torque of approximately 5–10 N·cm, as per manufacturer recommendations. The RFA probe was positioned at a standardized distance and orientation relative to the SmartPeg to minimize operator-induced variability. ISQ values were recorded in two orthogonal directions—buccolingual and mesiodistal—reflecting the mechanical response of the peri-implant bone to vibrational stimuli across both axial planes. Three repeated measurements were performed in each direction, and the mean of all six values was calculated to determine the final ISQ score for each implant. Measurements were conducted under sterile conditions with the animals maintained under general anesthesia to ensure precision and eliminate movement artifacts. ISQ values, expressed on a scale from 1 to 100, reflect the degree of micromotion at the implant–bone interface and serve as a surrogate marker of osseointegration, with higher values indicating greater stability. All measurements were performed by a single calibrated examiner to enhance consistency and minimize inter-operator variability.

### 2.3. Evaluation of the Bone–Implant Interface Gap by Micro-CT 3D Deviation

Following a 4-week healing period, the tibial segments containing the implants were carefully harvested and sectioned. Non-destructive evaluation of BIG was performed using a high-resolution micro-computed tomography (micro-CT) system (Quantum FX; PerkinElmer, Waltham, MA, USA) at an isotropic voxel size of 17.2 μm. Approximately 800 cross-sectional images were acquired per specimen, with each image having a resolution of 1024 × 1024 pixels.

**Figure 5:**
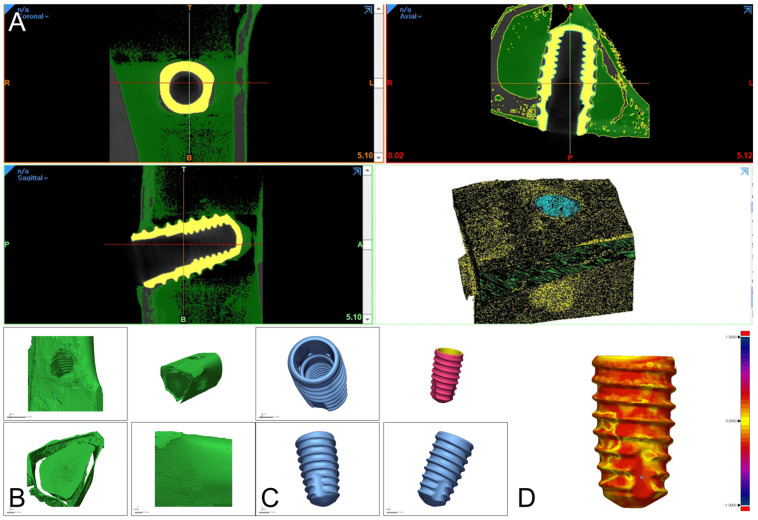
Three-dimensional analysis of the bone–implant interface gap using micro-computed tomography and 3D modeling software. (**A**) Segmentation of the implant and adjacent bone using Mimics software version 24.0 (Materialise, Leuven, Belgium), visualized in the axial, sagittal, and coronal planes. (**B**) Reconstructed 3D model of the tibial bone. (**C**) Segmented implant models visualized in Geomagic Control X (3D Systems, Rock Hill, SC, USA). (**D**) Quantitative assessment of the bone–implant interface gap (µm) via 3D deviation mapping (visual tolerance ±100 µm).

DICOM datasets were reconstructed into three-dimensional (3D) models using dedicated medical imaging software (Mimics Research version 24.0; Materialise, Leuven, Belgium). Segmentation of the titanium implant and surrounding bone tissue was performed using intensity thresholding, followed by manual refinement. Multiplanar reconstruction views (axial, coronal, and sagittal) were referenced simultaneously to ensure accurate delineation between cortical and trabecular bone structures and the implant surface (Figure 5A). The segmented geometries were subsequently converted into STL file format.

These STL files were then imported into industrial metrology software (Geomagic Control X version 2022.0.0; 3D Systems, Rock Hill, SC, USA) for quantitative analysis. Implant and bone models were registered using a best-fit alignment algorithm. BIG was evaluated by calculating the Euclidean distance between corresponding points on the implant and bone surfaces across the entire interface using point cloud-based 3D deviation analysis (Figure 5B–D).

The root mean square (*RMS*) error was computed to quantify the average interfacial gap using the following formula:

(1) $$RMS=\frac{1}{\sqrt{n}}\cdot \sqrt{\sum_{i=1}^{n}{\left(d_{i}\right)}^{2}}$$

where *d_i_* represents the distance between each pair of corresponding points on the implant reference model and the reconstructed tibial bone model, and *n* denotes the total number of data points analyzed. Lower *RMS* values indicate a more intimate and continuous bone-to-implant interface, reflecting superior osseointegration. To complement the quantitative analysis, color-coded deviation maps were generated to visualize the spatial distribution of interfacial conformity. For color-map visualization only (not for computing metrics), we used a tolerance band of ±0.10 mm (±100 µm), chosen to be physiologically meaningful and aligned with the voxel size (17.2 µm; ≈6 voxels) and typical trabecular dimensions. All analyses were performed by a single calibrated examiner, and intra-examiner reliability was confirmed through repeated segmentation of randomly selected specimens. In this study, the micro-CT interface gap is a geometric surrogate of interfacial conformity and is not equivalent to histologic bone-to-implant contact.

### 2.4. Evaluation of Removal Torque of Placed Implants

**Figure 6:**
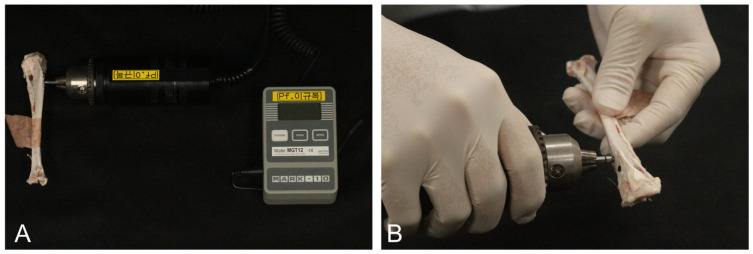
Measurement of implant removal torque in rabbit tibiae. (**A**) A digital torque gauge (Model MGT12, Mark-10 Corp., Copiague, NY, USA) was attached to the implant for measurement. (**B**) Maximum removal torque was recorded during manual counterclockwise rotation until full dislodgment of the implant.

To assess the mechanical stability of osseointegration, removal torque testing was performed using a digital torque gauge (Model MGT12; Mark-10 Corp., Copiague, NY, USA). Following euthanasia, the proximal tibial segments containing the implants were carefully harvested and debrided of soft tissue to preserve the integrity of the bone–implant complex and prevent contamination (Figure 6A).

Each specimen was rigidly secured in a custom-designed fixation jig to eliminate micromovements during testing. The torque driver was engaged with the internal connection of the implant, and a controlled counterclockwise rotation was manually applied until complete dislodgment of the implant from the surrounding bone was achieved (Figure 6B). The peak torque value (N·cm) required for complete removal was automatically recorded by the digital gauge.

All measurements were conducted under standardized ambient conditions by a single calibrated examiner. To minimize operator-induced variability, each removal torque test was repeated twice at 5 min intervals, and the mean of the two values was used for statistical analysis. The removal torque value served as a quantitative indicator of mechanical interlocking between the implant surface and newly formed bone, providing a surrogate measure of secondary implant stability.

### 2.5. Statistical Analysis

All analyses reflected the clustered, within-animal crossover design (four implants per rabbit). Continuous outcomes were modeled using linear mixed-effects models estimated by REML, with rabbit specified as a random intercept to account for intra-rabbit correlation. For the ISQ, observations at two time points (immediately after placement and at four weeks) were analyzed with fixed effects for group (NSK vs. SAESHIN), time, and their interaction. From these models, we report estimated marginal means (EMMs) with 95% confidence intervals (CIs) and two-sided *p*-values (α = 0.05) for planned contrasts (between-group differences at each time point and within-group change over time).

Removal torque and BIG (or interface-gap distance) were analyzed at retrieval with mixed models, including group as a fixed effect and rabbit as a random intercept; for region-specific BIG, region (proximal, distal) and group × region interaction were added as fixed effects. For all mixed models, we present variance components (between-rabbit and residual) and the corresponding intraclass correlation coefficient (ICC = σ^2^_rabbit/[σ^2^_rabbit + σ^2^_residual]) to quantify clustering. Degrees of freedom were obtained via Satterthwaite approximation.

Clinical equivalence at four weeks for ISQ was evaluated using the two one-sided test (TOST) applied to the mixed-model EMM difference (SAESHIN–NSK) with a pre-specified equivalence margin of ±5 ISQ units; results are summarized as 90% CIs for the EMM difference, together with one-sided *p*-values for both tests.

Immediate ISQ was treated as an exploratory endpoint reflecting stiffness under a standardized seating torque of 35 N·cm. Because insertion torque was fixed, immediate ISQ was not interpreted as device-dependent primary mechanical interlock. Primary inference focused on between-system differences at four weeks for ISQ, removal torque, and BIG. To examine the association between four-week ISQ and removal torque while respecting clustering, we fitted a mixed-effects regression with torque as the dependent variable, ISQ as a fixed effect, and rabbit as a random intercept; we report the slope estimate (β) with 95% CI, *p*-value, and ICC. Within-session repeatability of the ISQ measurements was summarized using ICC (3,1). Model assumptions were assessed by inspection of conditional residuals (Q–Q plots; residuals versus fitted values) and influence diagnostics. As part of sensitivity analyses, side (right/left) and implant position (anterior/posterior) were explored as fixed covariates to evaluate robustness without overfitting. Unadjusted independent/paired *t*-tests from preliminary analyses are provided only as supplementary sensitivity results and were not used for inference.

## 3. Results

### 3.1. Implant Stability

**Figure 7:**
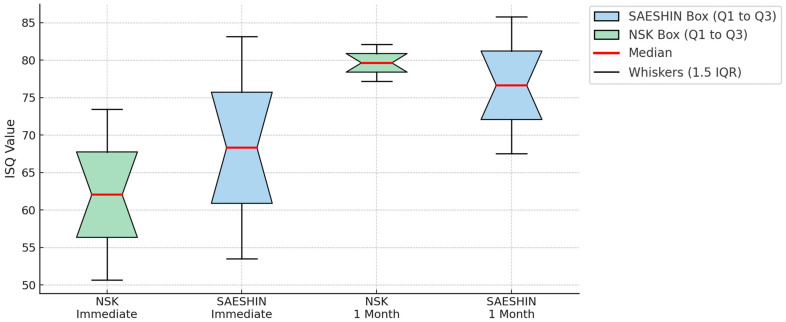
Comparison of implant stability quotient (ISQ) values between the NSK and SAESHIN groups at two time points. Boxplots show ISQ measurements obtained immediately after placement and at 1 month postoperatively. Boxes represent the interquartile range (IQR; Q1–Q3), red lines indicate the median, whiskers denote 1.5 × IQR. A significant difference was noted between groups at baseline (*p* < 0.001).

At baseline, under the fixed 35 N·cm seating torque, the SAESHIN system showed a higher adjusted ISQ than the NSK system. This difference reflects stiffness measured under a controlled torque condition rather than superiority in device-dependent primary mechanical interlock. Immediately after placement, the SAESHIN system yielded a higher adjusted ISQ than the NSK system (Table 1; Figure 7). The model-estimated between-group contrast at baseline was +6.9 ISQ units (SAESHIN–NSK; 95% CI +1.3–+12.5; *p* = 0.018). At 1 month, adjusted ISQ values did not differ between the systems (difference of −1.2 ISQ units; 95% CI −4.3–+1.9; *p* = 0.42), indicating comparable secondary stability. The group × time interaction term was statistically significant, consistent with a steeper ISQ gain for NSK across the healing interval (Table 1). Clustering by rabbit was non-negligible (intra-class correlation, ICC = 0.36; Table 2). Visual inspection of Figure 7 aligns with these inferences, showing wider dispersion immediately after placement and convergence at 1 month.

### 3.2. Removal Torque

**Figure 8:**
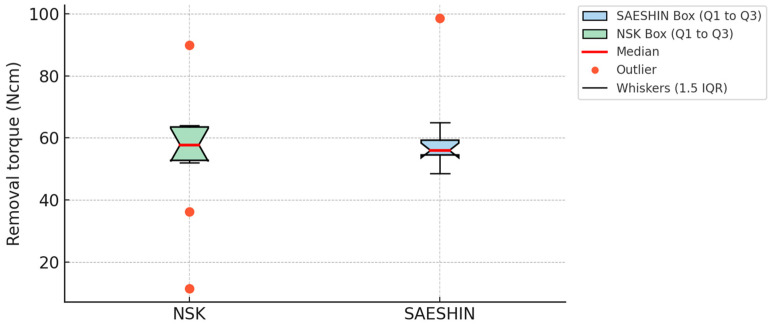
Comparison of implant removal torque values between the NSK and SAESHIN groups. Boxplots display removal torque values (N·cm) at the time of implant retrieval. Boxes represent the interquartile range (IQR; Q1–Q3), red lines indicate the median, whiskers denote 1.5 × IQR, and orange dots represent outliers.

At 1 month, adjusted mean removal torque did not differ by system (Table 3; Figure 8). The LMM estimated a contrast of +4.3 N·cm (SAESHIN–NSK; 95% CI −5.2–+13.8; *p* = 0.36). Rabbit-level clustering accounted for approximately one-third of the variance (ICC = 0.31; Table 2).

### 3.3. Bone–Implant Interface Gap (Micro-CT 3D Deviation)

**Figure 9:**
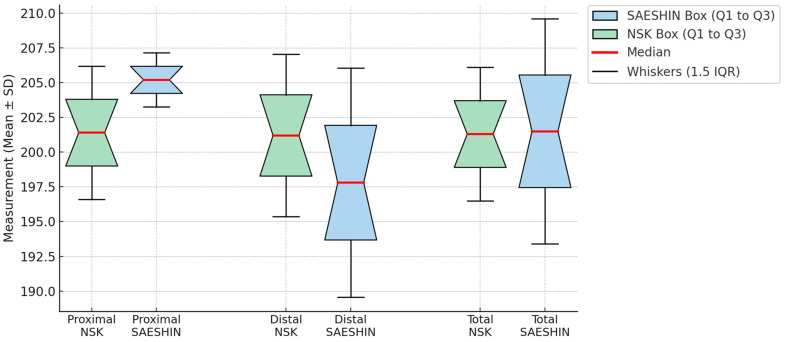
Comparison of bone–implant interface gap (BIG) values between the NSK and SAESHIN groups by anatomical region. Boxplots show BIG measurements at the proximal, distal, and total regions. Boxes represent the interquartile range (IQR; Q1–Q3), red lines indicate the median, whiskers denote 1.5 × IQR, and orange dots represent outliers.

**Figure 10:**
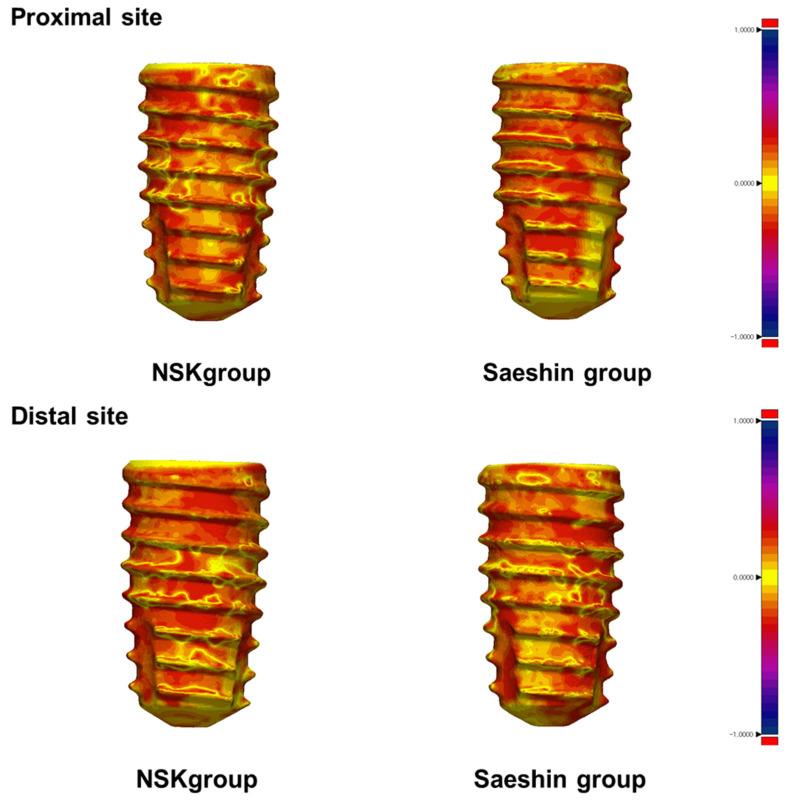
Three-dimensional deviation maps of bone–implant interface gap surfaces in the NSK and SAESHIN groups. Representative color-coded maps illustrate the interfacial gap between the implant and surrounding bone at proximal and distal sites. The color scale indicates deviation magnitude (in millimeters), with red denoting larger separations and yellow indicating closer apposition.

Quantitative BIG metrics showed no statistically significant between-system differences at the proximal, distal, or total regions (Table 4; Figure 9). Model-estimated contrasts (SAESHIN–NSK) were +3.3 µm at the proximal site (95% CI −0.6–+7.2; *p* = 0.10), −2.6 µm at the distal site (95% CI −7.0–+1.8; *p* = 0.24), and +0.3 µm for the total measure (95% CI −3.5–+4.1; *p* = 0.88). BIG exhibited minimal clustering (ICC = 0.07; Table 2). Qualitative 3D deviation maps (Figure 10) corroborate the quantitative results, demonstrating generally favorable thread-level adaptation in both systems with only minor, spatially localized deviations.

### 3.4. Equivalence Testing, Variance Components, and Ancillary Association

Clinical equivalence of 1-month ISQ between the systems, evaluated using the two one-sided test (TOST) procedure with a pre-specified margin of ±5 ISQ units, is supported (Table 5; *p*_lower = 0.024; *p*_upper = 0.019, both <0.05). Variance components and ICCs for each endpoint are summarized in Table 2, underscoring the necessity of modeling within-rabbit correlation. An ancillary, implant-level partial correlation (controlling for rabbit via random intercepts) indicates a moderate positive association between 1-month ISQ and removal torque (r_partial = 0.41; 95% CI 0.01 to 0.68; *p* = 0.041; Table 6), consistent with the expectation that greater secondary stability corresponds to stronger mechanical interlock.

## 4. Discussion

This study evaluated and compared two surgical micromotor systems, a conventional micromotor system (NSK SurgicPro+) and a diode laser-integrated micromotor system (Saeshin BLP 10), with respect to implant stability, BIG, and removal torque in a within-animal crossover rabbit tibia model. Linear mixed-effects analyses that accounted for within-rabbit clustering showed no statistically significant between-system differences in secondary stability, BIG, or removal torque (*p* > 0.05), whereas the diode laser-integrated micromotor system showed higher immediate ISQ under a fixed 35 N·cm seating torque (*p* < 0.05). This early difference should be interpreted as stiffness under standardized torque and not as evidence of greater device-dependent primary mechanical interlock.

Immediate ISQ under standardized torque is a key determinant of early osseointegration and subsequent implant success. The higher immediate postoperative ISQ in the SAESHIN group indicates an advantage in initial mechanical anchorage. This finding is in accordance with reports that emphasize the role of surgical instrumentation in optimizing immediate ISQ under standardized torque [32,33,34,35,36]. In particular, diode laser-integrated micromotor systems that maintain accurate torque delivery and improve thermal control can mitigate drilling-related heat and microdamage, thereby supporting early stability [37,38]. Clinically, a higher baseline ISQ may support cautious consideration of immediate loading in carefully selected indications; however, interpretation should be integrated with insertion torque, local bone quality, and prosthetic risk. Given that insertion torque was standardized at 35 N·cm in this model and the between-group difference attenuated by four weeks, the early advantage should not be construed as evidence of superior secondary stability. Verification in prospective clinical studies with predefined immediate loading protocols is required to determine whether the observed difference translates into a clinically meaningful benefit.

Despite the early advantage in immediate ISQ under standardized torque observed for the SAESHIN micromotor system, adjusted ISQ values converged by the four-week assessment, indicating no between-system difference in secondary stability and supporting equivalence within the prespecified ±5 ISQ margin. This trajectory is in accordance with prior reports showing that advanced instrumentation can improve initial mechanical anchorage without conferring durable gains in implant fixation [39,40,41]. Taken together, the evidence highlights the multifactorial nature of osseointegration, arising from the interactions among drilling mechanics, implant macro- and microgeometry, surface characteristics, local bone quality, and host remodeling biology.

Removal torque testing, which reflects mechanical retention at the bone–implant interface, was concordant with the ISQ findings and supported equivalence of the two micromotor systems at four weeks. The linear mixed-effects model estimated a small, non-significant advantage for the SAESHIN micromotor system (adjusted mean difference +4.3 N·cm; 95% CI −5.2–+13.8; *p* = 0.36). This pattern aligns with prior reports indicating that modest differences in surgical instrumentation rarely produce meaningful gains in ultimate osseointegration when biological conditions are favorable [42,43]. The observed dispersion likely reflects interindividual variation in bone quality, early remodeling dynamics, and local anatomic context, recognized sources of heterogeneity in preclinical models [44,45,46].

Quantitative assessment of bone–implant interface gap using high-resolution micro-computed tomography with three-dimensional deviation mapping showed no statistically significant differences between systems across proximal, distal, or total regions (Table 4; Figure 9 and Figure 10). Model-estimated contrasts were small, with 95% CIs spanning zero, and clustering by rabbit was minimal for BIG (intraclass correlation coefficient 0.07; Table 2). Although focal deviations were qualitatively visible in some SAESHIN specimens near the apex and within the thread valleys, these features did not affect the overall BIG metrics. Such site-specific patterns are more plausibly explained by local surgical factors, measurement variability, or early remodeling than by systematic effects of the micromotor system [47,48].

Use of micro-CT-based three-dimensional modeling enabled high-resolution, non-destructive characterization of peri-implant bone adaptation and interfacial conformity. Standardized segmentation, best-fit registration, and color-coded deviation mapping have been validated for precision and reproducibility, and their application in this study strengthens internal validity and complements the mechanical endpoints [49,50].

A statistically significant group × time interaction indicated a steeper ISQ increase over four weeks for the NSK micromotor system relative to the diode laser-integrated micromotor system. This pattern is biologically plausible: differences in initial bone compression or microtrauma during osteotomy and seating can transiently depress early stability and subsequently drive remodeling-related gains. Prior work has shown that the magnitude and distribution of early surgical trauma modulate the temporal trajectory of implant stability, aligning with the observed system-specific profiles [51,52].

The correlation analysis between four-week ISQ and removal torque supports the clinical utility of ISQ as a surrogate of interfacial strength. After accounting for within-rabbit clustering, a moderate positive association was observed (partial r = 0.41; 95% CI 0.01–0.68), consistent with reports that resonance frequency analysis provides a noninvasive predictor of implant stability [53,54]. A moderate, rather than strong, correlation is mechanistically expected because the two measures interrogate related but distinct constructs: ISQ reflects axial stiffness under small-amplitude vibration, whereas removal torque quantifies peak interfacial shear strength at failure. Site-level heterogeneity in cortical thickness, trabecular density and orientation, and early remodeling at four weeks can differentially influence stiffness and shear. Procedural and measurement variability, including osteotomy heat or microdamage, minor seating-depth differences, SmartPeg tightening and orientation, and the torque-testing rate and lever arm, add further dispersion. Range restriction from the standardized 35 N·cm insertion torque and residual within-animal clustering may also attenuate the observable association. Taken together, these factors support interpreting ISQ alongside insertion torque and complementary clinical or radiographic indicators rather than as a stand-alone determinant.

Recent biomechanical investigations provide additional context for these findings. Digital image correlation studies indicate that implant material influences peri-implant stress distribution, with zirconia and titanium producing distinct cortical strain profiles [55]. Finite element analyses of orthopedic knee components likewise show that, even with identical macrogeometry, the metallic alloy selected alters load transfer and the potential for peri-prosthetic remodeling [56]. Extending these principles to dental implants, parametric designs with graded internal porosity have been shown to homogenize peri-implant stress fields and may accelerate early osseointegration [57]. Taken together, this literature supports a systems view in which optimal outcomes arise from the combined effects of surgical instrumentation, implant material, and macro- and micro-structural design, each shaping the mechanical milieu that governs bone healing.

Several limitations should be acknowledged. The four-week observation window precluded assessment of long-term remodeling, functional loading, and late complications. The sample size was modest and confined to a single species and anatomical site, which limits external generalizability. Standardization of insertion torque at 35 N·cm, use of one implant macro-design, and reliance on a single RFA platform may also have attenuated between-system differences. Although a within-animal crossover design and mixed-effects modeling were used to address clustering, the study was not powered to examine interactions with bone quality, limb side, or position.

Within these constraints, the comparative analysis indicated that the diode laser-integrated micromotor system achieved higher immediate ISQ under standardized torque at placement, whereas secondary stability at four weeks, removal torque, and micro-CT-derived bone–implant interface gap did not differ from the conventional micromotor system. Three-dimensional deviation mapping supported these results by showing no systematic regional discrepancies in interfacial conformity. Taken together, the data support practical interchangeability of the two systems for early osseointegration end points under standardized conditions.

Caution is warranted when extrapolating these findings to human clinical practice. Confirmation in adequately powered clinical trials with longer follow-up, diverse jaw sites and bone densities, direct thermal monitoring during osteotomy, and preregistered equivalence margins is recommended. Inclusion of patient-centered outcomes and immediate or early loading protocols will be important to determine whether an early difference in immediate ISQ under standardized torque translates into clinically meaningful benefit.

## 5. Conclusions

Within the limits of this within-animal crossover rabbit tibia study, the diode laser-integrated micromotor system achieved a higher immediate ISQ under standardized 35 N·cm seating torque compared with the conventional micromotor system. By four weeks, no differences were observed between the systems in ISQ, removal torque, or micro-CT–derived BIG, and equivalence in ISQ was confirmed. These findings suggest that micromotor selection does not materially influence early osseointegration under standardized conditions, supporting their practical interchangeability during the initial healing phase. The transient advantage in primary stability with the diode laser-integrated system likely reflects stiffness under fixed torque rather than superior device-dependent interlocking. Further well-designed clinical trials with longer follow-up and diverse bone conditions are warranted to determine whether these preclinical observations translate into clinically relevant benefits.

## Figures and Tables

**Table 1:** Adjusted implant stability by micromotor system and time from linear mixed-effects models.

Outcome/Time	NSK, Adjusted Mean (95% CI)	SAESHIN, Adjusted Mean (95% CI)	Difference (SAESHIN–NSK)	*p*-Value	Group × Time *p*
ISQ, immediate	64.8 (61.0–68.6)	71.7 (67.9–75.6)	+6.9 (+1.3–+12.5)	0.018	0.011
ISQ, 1 month	79.8 (77.7–81.9)	78.6 (76.4–80.8)	−1.2 (−4.3–+1.9)	0.42	

LMM with fixed effects for group (NSK vs. SAESHIN), time (immediate vs. 1 month), and group × time interaction; random intercept for rabbit. Restricted maximum likelihood estimation; Satterthwaite degrees of freedom. Values are marginal means (95% CI). Two-sided α = 0.05. Immediate ISQ measured under standardized 35 N·cm seating torque; not interpreted as device-dependent primary mechanical interlock.

**Table 2:** Variance components and intra-class correlation coefficients (ICC) from mixed models.

Outcome	σ^2^_Rabbit (SE)	σ^2^_Residual (SE)	ICC
ISQ (both times)	22.8 (8.1)	40.7 (7.5)	0.36
Removal torque	68.5 (24.3)	152.0 (31.8)	0.31
BIG (total)	3.2 (1.9)	43.7 (8.6)	0.07

ICC = σ^2^_rabbit/(σ^2^_rabbit + σ^2^_residual). Variance components from REML LMMs specified in Table 1, Table 2 and Table 3.

**Table 3:** Adjusted removal torque (N·cm) at 1 month from linear mixed-effects models.

Outcome	NSK, Adjusted Mean (95% CI)	SAESHIN, Adjusted Mean (95% CI)	Difference (SAESHIN–NSK)	*p*-Value
Removal torque (N·cm)	55.8 (47.4–64.2)	60.1 (52.5–67.7)	+4.3 (−5.2–+13.8)	0.36

LMM with fixed effect for group and random intercept for rabbit (REML; Satterthwaite df). Two-sided α = 0.05.

**Table 4:** Adjusted bone–implant interface gap deviation (µm) by region from linear mixed-effects models.

Region	NSK, Adjusted Mean (95% CI)	SAESHIN, Adjusted Mean (95% CI)	Difference (SAESHIN–NSK)	*p*-Value
Proximal	201.6 (199.7–203.5)	204.9 (203.2–206.7)	+3.3 (−0.6–+7.2)	0.10
Distal	201.0 (198.6–203.4)	198.4 (195.9–200.8)	−2.6 (−7.0–+1.8)	0.24
Total	201.3 (199.5–203.0)	201.6 (199.7–203.4)	+0.3 (−3.5–+4.1)	0.88

Region-specific LMMs with fixed effect for group and random intercept for rabbit (REML; Satterthwaite df). Two-sided α = 0.05.

**Table 5:** Equivalence testing (TOST) for 1-month ISQ, with ±5 ISQ units as the clinical equivalence margin.

Contrast	Equivalence Margin	*p*_Lower	*p*_Upper	Conclusion
SAESHIN–NSK at 1 month	±5 ISQ	0.024	0.019	Equivalent (both *p* < 0.05)

TOST performed on the LMM-estimated 1-month group contrast. Equivalence concluded when both one-sided tests against the lower (−5) and upper (+5) bounds were significant at α = 0.05.

**Table 6:** Ancillary association between 1-month ISQ and removal torque (implant-level partial correlation controlling for rabbit).

Correlation	r_Partial (95% CI)	*p*-Value
ISQ (1 month) vs. removal torque	0.41 (0.01–0.68)	0.041

Pearson correlation on implant-level values residualized for rabbit via random intercepts; inference via parametric bootstrap. Not adjusted for multiplicity.

## Data Availability

The data supporting the findings of this study are available from the corresponding author upon reasonable request. Due to privacy and proprietary restrictions, the datasets are not publicly accessible.
